# ﻿Annotated catalogue of Orthoptera (Orthoptera, Insecta) of Latvia

**DOI:** 10.3897/zookeys.1134.95637

**Published:** 2022-12-05

**Authors:** Rūta Starka, Uģis Piterāns, Voldemārs Spuņģis

**Affiliations:** 1 Department of Zoology and Animal Ecology, University of Latvia, Riga, Latvia University of Latvia Riga Latvia; 2 Zoology Department, Latvian National Museum of Natural History, Riga, Latvia Zoology Department, Latvian National Museum of Natural History Riga Latvia

**Keywords:** Baltics, citizen science, diversity, faunal checklist, historic review

## Abstract

We present a revised list of Latvian species of Orthoptera and provide notes on their occurrence and present knowledge. New information on orthopteran observations from online databases, local unpublished studies, entomological collections, and our direct observations is combined, and a dataset of more than 1500 recent observations is provided. All historical synonyms used in the reviewed information sources are presented. As a result, an annotated list of 52 Orthoptera species is compiled, from which five newly reported species in Latvia are presented here for the first time together with distribution maps. In conclusion, the presence of 43 species of Orthoptera is confirmed in Latvia.

## ﻿Introduction

The first mentions of orthopteroids in the territory of Latvia date back to 18^th^ century (Fischer 1778), shortly after the work of C. Linnaeus (Linnæus 1758). At that time, they were treated as a part of Hemiptera, and the first existing list of species contains only nine orthopteroid species (Fischer 1778). Later Orthoptera was treated as a separate taxonomic group which included at that time earwigs (Dermaptera) and cockroaches (Blattodea) (Kawall 1864). At this point, 23 species were already listed in Courland’s (western Latvia) fauna. The first thorough review of Latvian Orthoptera fauna was published in 1943 by the renowned Latvian entomologist Kārlis Princis. At that time Dictyoptera (cockroaches, termites, and mantises) was a suborder of Orthoptera (Princis 1943), until 1979, when the Dictyoptera were removed and treated as a separate order (Miskelly and Paiero 2019). Therefore, while K. Princis (1943) mentioned 54 orthopteran species in Latvia, nowadays it corresponds to only 43 species. After K. Princis left Latvia in 1944 to continue his research on cockroaches (Blattaria) in Sweden (Gurney et al. 1979), fundamental faunal research of Orthoptera in Latvia stopped.

With the growing popularity of citizen science (hereafter referred to as “CS”) platforms and successful nominations of some Orthoptera species as “Insect of the Year” by The Entomological Society of Latvia (*Psophusstridulus* in 2001, *Achetadomesticus* in 2002, *Gryllotalpagryllotalpa* in 2007, and *Oedipodacaerulescens* in 2013), the interest of Orthoptera and other insects has grown and resulted in accumulated, unpublished observational data on CS platforms. In Latvia, the foremost popular and most commonly used CS platform is “Dabasdati.lv” (Latvijas Dabas Fonds and Latvijas Ornitoloģijas biedrība 2021). This database was developed in 2008 by the Latvian Fund for Nature and the Latvian Ornithological Society with the aim to develop a volunteer-based online database where records of any species can be uploaded and pinned to coordinates. After the upload of the observation, the species record is revised by a group of experts, similar as done by “iNaturalist” (California Academy of Sciences 2021). Examples where such CS platforms have proven to give crucial information on distribution and occurrence of species are numerous (Chandler et al. 2017; Moulin 2020). Therefore, it is important to summarize and publish the data obtained on local CS platforms, to ensure knowledge transfer internationally.

Until now, there are a few lists of Latvian Orthoptera, each including up to 45 species. However, none of these lists are annotated, nor do they critically review the historic data, and they are not taxonomically up-to-date. Now, at a time of great declines of biodiversity, it is important to summarize and update information on Latvian Orthoptera to set a new baseline after almost 80 years since the last thorough review. Therefore, the aim of this study is to create a revised, annotated list of Orthoptera species in Latvia and to discuss their distribution and occurrence. To do so, we update the scientific nomenclature to the latest taxonomic changes, review all historical records of each species, gather recent unpublished data, and compile the latest occurrences and habitat preferences in Latvia of each species.

## ﻿Material and methods

### ﻿Territory and habitats

Latvia is in the center of the Baltic region, situated between Lithuania in the south and Estonia in the north, and occupies a total area of 64573 km^2^, from which 62210 km^2^ are land areas (Kūle 2021). The climate in Latvia is significantly influenced by the proximity of the Atlantic Ocean and the long coastline with the Baltic Sea, that determine the domination of cyclonic activity. The mean annual air temperature varies between 5.2–7.4 °C (mean diurnal temperatures 18.8–16.8 °C in July and −1.6 to −5.8 °C in February), and the mean annual precipitation is 683 mm (Briede 2021). Latvia is in the boreonemoral biome, and, therefore, the final phase of natural succession in most terrestrial habitats, if not managed or disturbed, is forest (Priede 2017).

Grassland, dune, heathland, and mire habitats are important for orthopteran diversity in Latvia (Spuņģis 2007, 2013; Rozenfelde et al. 2017; Rozenfelde 2018; Rūsiņa 2020). The majority of grasslands in Latvia are cultivated, and only ~0.7% of the country’s territory is occupied by natural or seminatural grasslands (Rūsiņa 2020) which can be categorized in 10 EU-protected habitat types (Rūsiņa 2013). EU-protected coastal and inland dune habitats make up ~1% of the territory (Rove 2013a), from which secondary dunes (grey and brown dunes) are particularly valuable to orthopteran diversity (Spuņģis 2007). European dry heaths hold a great conservation value and are even rarer than dunes in Latvia (Rove 2013b; Rozenfelde 2018). Mire habitats occupy roughly 5% of the country’s territory, and, due to specific environmental conditions, is inhabited by specialized, often rare species (Auniņa 2013). There are 333 Natura 2000 sites in Latvia, of which terrestrial sites make up 12% of the land area (Dabas aizsardzības pārvalde 2021).

### ﻿Data resources

The species list was created by adding up all the available information from historical records (Fischer 1778; Kawall 1864; Princis 1931, 1932, 1933, 1934a, 1934b, 1935, 1936, 1939, 1943; Spuris 1957, 1998; Ozols 1963), species lists (Heller et al. 1998; Spuņģis and Kalniņš 2002; Matisons 2004; Willemse and Heller 2013), reports of new species (Gailis et al. 2003; Sokolovskis and Suveizda 2012), the entomological collection of the Latvian National Museum of Natural History (LMNH), and previously unpublished data of the observations of new species in Latvia (Latvijas Dabas Fonds and Latvijas Ornitoloģijas biedrība 2021).

Taxonomical hierarchy was obtained from the “Orthoptera Species File” online database (Cigliano et al. 2022). Synonyms from the reviewed literature are provided. If the presence of the species in Latvia is doubtful, but possible, the symbol “(?)” was used in front of the species name. Similarly, if the species presence is insufficiently proven the symbol “(–)” was used.

Notes on occurrence in Latvia were combined from original data of the online databases “Dabasdati.lv” (Latvijas Dabas Fonds and Latvijas Ornitoloģijas biedrība 2021), “iNaturalist” (California Academy of Sciences 2021) and local ecological studies (Matisons 2005; Spuņģis 2007, 2013; Rozenfelde et al. 2017; Rozenfelde 2018). In this article, a species observation from CS record was used only if a photo of the species was provided together with the coordinates, or if the observation was made by a biologist with experience in insect identification. The occurrence and distribution information from “Dabasdati.lv” was interpreted with caution, as these data are not obtained by systematic research, and the number of observations is higher near large cities where more people live. Because some data may be transferred from one platform to another, a manual cross-reference was carried out to avoid doubling up of data. For “iNaturalist” data, the “Verifiable Observations” filter was used. For many species of Acrididae, data in CS platforms are scarce or lacking due to complicated or impossible species determination from photos. Therefore, some notes on distribution and habitats are added from our own observations. For species that are newly reported from the Latvian Orthoptera fauna, distribution and range maps were created in ArcMap (ArcGIS Desktop v. 10.6), using ETRS89 LAEA Europe coordinate system. A 10 × 10 km grid was intersected with the observation data (Suppl. material 1) to create distribution maps. Then, similarly to the Reporting Guidelines for Article 17 of the EU Habitats Directive methodology (DG Environment 2017), a 40 km buffer was created around each observation point to create range maps.

## ﻿Results

**Figure 1.**
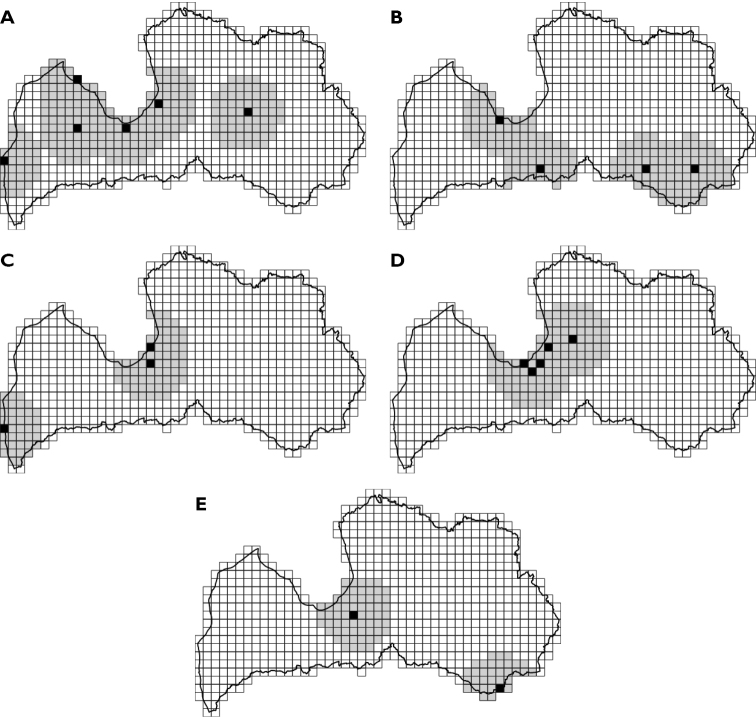
Distribution of five new species to Latvia. Observations are shown on 10 × 10 km grid cells. Black cells indicate distribution derived from observations. Gray cells indicate possible range (40 km buffer from each observation point) **A***Chorthippusvagans***B***Conocephalusfuscus***C***Leptophyespunctatissima***D***Meconemathalassinum***E***Bicoloranabicolor*.

When combining all the available information on the Latvian orthopteran fauna, a list of 52 species belonging to 34 genera and six families was obtained. From the analyzed species, five are newly reported from Latvia (Fig. 1A–E, Table 1). Overall 43 species are with more-or-less certain occurrence (Table 1), but the presence of nine species is doubtful and not proven; therefore, these nine species should be excluded from the list of Orthoptera in Latvia. A more detailed analysis of all 52 species, the history of their inclusion in the Orthoptera fauna in Latvia, and historically used synonyms, as well as known information on occurrence, conservation status, and habitat preference is available in Suppl. material 2.

**Table 1.** Check-list of Orthoptera species in Latvia. Newly reported species are indicated with an asterisk (*).

Suborder	Family	Subfamily	Species
Caelifera	Acrididae MacLeay, 1821	Gomphocerinae Fieber, 1853	Chorthippus (Chorthippus) albomarginatus (De Geer, 1773)
			Chorthippus (Chorthippus) dorsatus (Zetterstedt, 1821)
			Chorthippus (Glyptobothrus) apricarius (Linnaeus, 1758)
			Chorthippus (Glyptobothrus) biguttulus (Linnaeus, 1758)
			Chorthippus (Glyptobothrus) brunneus (Thunberg, 1815)
			Chorthippus (Glyptobothrus) pullus (Philippi, 1830)
			Chorthippus (Glyptobothrus) vagans (Eversmann, 1848)*
			*Chrysochraondispar* (Germar, 1834)
			*Euthystirabrachyptera* (Ocskay, 1826)
			*Myrmeleotettixmaculatus* (Thunberg, 1815)
			Omocestus (Omocestus) haemorrhoidalis (Charpentier, 1825)
			Omocestus (Omocestus) viridulus (Linnaeus, 1758)
			*Pseudochorthippusmontanus* (Charpentier, 1825)
			*Pseudochorthippusparallelus* (Zetterstedt, 1821)
			*Stauroderusscalaris* (Fischer von Waldheim, 1846)
			*Stenobothruslineatus* (Panzer, 1796)
			*Stenobothrusstigmaticus* (Rambur, 1838)
		Melanoplinae Scudder, 1897	*Podismapedestris* (Linnaeus, 1758)
		Oedipodinae Walker, 1871	*Locustamigratoria* (Linnaeus, 1758)
			*Oedipodacaerulescens* (Linnaeus, 1758)
			*Psophusstridulus* (Linnaeus, 1758)
			Sphingonotus (Sphingonotus) caerulans (Linnaeus, 1767)
			*Stethophymagrossum* (Linnaeus, 1758)
	Tetrigidae Rambur, 1838	Tetriginae Rambur, 1838	*Tetrixbipunctata* (Linnaeus, 1758)
			*Tetrixsubulata* (Linnaeus, 1758)
			*Tetrixtenuicornis* (Sahlberg, 1891)*
			*Tetrixundulata* (Sowerby, 1806)
Ensifera	Gryllidae Laicharting, 1781	Gryllinae Laicharting, 1781	*Achetadomesticus* (Linnaeus, 1758)
	Gryllotalpidae Leach, 1815	Gryllotalpinae Leach, 1815	*Gryllotalpagryllotalpa* (Linnaeus, 1758)
	Rhaphidophoridae Walker, 1869	Aemodogryllinae Jacobson, 1905	Tachycines (Tachycines) asynamorus Adelung, 1902
	Tettigoniidae Krauss, 1902	Conocephalinae Kirby & Spence, 1826	Conocephalus (Anisoptera) dorsalis (Latreille, 1804)
			Conocephalus (Anisoptera) fuscus (Fabricius, 1793)*
		Meconematinae Burmeister, 1838	*Meconemathalassinum* (De Geer, 1773)*
		Phaneropterinae Burmeister, 1838	*Barbitistesconstrictus* Brunner von Wattenwyl, 1878
			*Leptophyespunctatissima* (Bosc, 1792)*
			Phaneroptera (Phaneroptera) falcata (Poda, 1761)
		Tettigoniinae Krauss, 1902	*Bicoloranabicolor* (Philippi, 1830)
			*Decticusverrucivorus* (Linnaeus, 1758)
			*Metriopterabrachyptera* (Linnaeus, 1761)
			*Pholidopteragriseoaptera* (De Geer 1773)
			*Roeselianaroeselii* (Hagenbach, 1822)
			*Tettigoniacantans* (Fuessly, 1775)
			*Tettigoniaviridissima* (Linnaeus, 1758)

## ﻿Discussion

Local faunal inventories are as important as ecological studies from a biogeographical viewpoint. Compiling all historical information shows how the fauna is changing with climate and how the knowledge of taxonomy and diversity has improved with time. According to the last IUCN Red List assessment of Orthoptera, 1082 species are native to or naturalised in Europe (Hochkirch et al. 2016a). Of these, 43 species (Table 1) are present in Latvia. While the proportion (4%) is not significant, we must consider that some of these species are on the border of their area of distribution. Our findings in this study clearly show that even if the local Orthoptera fauna is not rich in comparison to other insect orders, or compared with that of other European countries, there are some distribution and conservation issues to be dealt with.

Taxonomic changes in genera and species can be easily tracked using regularly updated databases such as Orthoptera Species File (Cigliano et al. 2022). Some unique synonyms were briefly used by Princis in the past for three species: *Metriopteragrisea* for the present-day *Platycleisgrisea* (Princis 1931), Stauroderus (Chorthippus) longicornis for *Pseudochorthippusmontanus* (Princis 1934b), and *Stauroderusparallelus* (Princis 1934b) and Stauroderus (Chorthippus) parallelus (Princis 1935) for *Pseudochorthippusparallelus*. It is important to summarize all possible synonyms to avoid confusion and misinterpretation when analysing historic data in the future.

Some of the species have problematic population status. For example, *Bryodemellatuberculata* is not only locally extinct in Latvia (Spuris 1998; Zuna-Kratky et al. 2016), but also extremely rare and vulnerable in all of Europe (Zuna-Kratky et al. 2016). The main known area of distribution of this species is south-western Russia, with subpopulations in the Baltics and Germany (Cigliano et al. 2022). Clearly there are vast geographical gaps between these populations, and this contributes to the difficulties of conservation. In the IUCN Red List, *B.tuberculata* is listed as Vulnerable in Europe, and due to a large-scale habitat deterioration, the population trend is decreasing (Zuna-Kratky et al. 2016). The species was recently rediscovered in Lithuania at one historical and one new locality (Budrys et al. 2008; Ūsaitis 2019), which might suggest that with proper habitat management and research effort, the *B.tuberculata* population could be re-established in Latvia. However, with the changes in the Cabinet of Ministers regulations (Ministru kabinets 2004a), the species has already been removed from the protected species list in Latvia, together with two other species of the subfamily Oedipodinae: *Sphingonotuscaerulans* and *Psophusstridulus*. *Sphingonotuscaerulans*, a species that has somewhat similar habitat requirements as other Oedipodinae, is extremely rarely found in Latvia, and, since 2004, it is no longer a protected species (Ministru kabinets 2004a). Very little is known about the distribution, occurrence, and sustainability of the population of this species. We suggest that *S.caerulans* might be at risk of local extinction. *Grylluscampestris* is another locally extinct species. In general, *G.campestris* is rare in central Europe, and while it is supposed to have stable population dynamics in north-eastern Europe, natural populations have been poorly investigated (Panagiotopoulou et al. 2016). This is also true for Latvia. In the list of European Orthoptera, the only countries or regions where *G.campestris* is considered absent is Finland, Estonia, Latvia, and northern Russia (Heller et al. 1998).

The examples above highlight the necessity of conservation actions. First of all, distributional studies are needed—to this day, no species of Orthoptera are monitored by any monitoring programme. From the available occurrence data, a number of species (e.g., *Myrmeleotettixmaculatus*, *Tetrixbipunctata*, *Pholidopteragriseoaptera*, *Conocephalusdorsalis*, and species of the subfamily Oedipodinae) show a coastal distribution pattern (Suppl. material 2). This can be explained by their habitat requirements, which mostly include some rare habitat types like dry heathlands, grey dunes, and calcareous fens along the Baltic Sea coast. In Latvia, three laws are instrumental to the protection of the species inhabiting the Baltic Sea and Riga Gulf coastlines: the Protection Zone Law (Saeima 1997), the Law on the Conservation of Species and Biotopes (Saeima 2000), and the Law on Specially Protected Nature Territories (Augstākā Padome 1993). However, with a monitoring programme, more information could be obtained on the distribution on these species and the efficiency of these laws. Secondly, conservation status needs to be assigned to more orthopteran species to justify conservation efforts. Today, only two species—*Podismapedestris* and *Oedipodacaerulescens*—are protected in Latvia (Ministru kabinets 2004b). Finally, conservation actions, such as habitat management and restoration, need to take place targeting these species.

The necessity of monitoring also applies to more common and new to Latvia species. For example, the geographical range borders of *Tetrixtenuicornis* are Spain in the south and Finland in the north (Hochkirch et al. 2016d), and the species is listed in the neighbouring Lithuanian Orthoptera checklist (Budrys and Pakalniškis 2007). Therefore, while there are few reliable records of this species in Latvia, it is expected to be found more commonly. Similarly, *Platycleisalbopunctata* is also listed in Lithuanian fauna, but seems to be restricted to the south of the country and the Baltic Sea coastline (Budrys and Pakalniškis 2007; California Academy of Sciences 2021). The population trend of this species is overall increasing, and it is expanding its range to the north (Hochkirch et al. 2016c). A newly reported species for Latvia, *Leptophyespunctatissima*, is a widespread species throughout western Europe, England, and southern Scandinavia (Cigliano et al. 2022), but the occurrence in the Baltics or Finland in unclear. This species has reduced wings in both sexes, but it has been presumed that relocation of individuals occurs via human transport (Hochkirch et al. 2016b). *Conocephalusfuscus* is also known to recently expand its distribution area to the north (Beckmann et al. 2015), while *Meconemathalassinum* is already on its northern border of distribution (Hochkirch et al. 2016e). Climate modelling research conducted in Russia predicted that *Calliptamusitalicus* will expand its range to the north (Popova et al. 2016). This species is present in Belarus (California Academy of Sciences 2021; Prischepchik and Storozhenko 2019) but not in Lithuania (Budrys and Pakalniškis 2007; California Academy of Sciences 2021). Today, *C.italicus* is remains distributed in southern Europe and is unlikely to be found in Latvia, except cases of accidental immigration of some individuals. Therefore, we can expect that with time the above-mentioned species, with the exception of *C.italicus*, could become more common or more commonly observed in Latvia.

With a warming climate, the dispersal of species to the north (Poniatowski and Fartmann 2011) will change the local fauna over time, and the arrival and disappearance of species is expected. An example of this is the first arrival of *Phaneropterafalcata* in Daugavpils in 2011 (Sokolovskis and Suveizda 2012) and its observation a year later approximately 230 km north-west from where it was first observed (unpublished data 2013, Ādaži military polygon). Today, *P.falcata* is a common species. Recently three additional species have been recorded in Lithuania (Ferenca et al. 2017; Budrys et al. 2019). While one of them, *Euthystirabrachyptera*, is already fairly common in Latvia, the other two species are yet to be found. In 2019, *Aiolopusthalassinus* was first recorded in Nida (only about 100 km south of the Latvian border), and the authors note that this could be yet another example of climate-change driven geographic range expansion to the north (Budrys et al. 2019). *Myrmecophilusacervorum* was found only about 70 km south of the Latvian border, in dead wood colonised by ants (Ferenca et al. 2017). Interestingly, Princis (1931) also mentioned this species as potentially present in Latvia. Kawall (1864) at his time also named 10 additional species that he thought could potentially be present in Latvia, and only one of those species—*Bohemanellafrigida*, referred to as *Pezotettixfrigida* by Kawall—has not been recorded to this date.

CS platforms, while being extremely useful, are not a substitute to monitoring, as the data obtained from them can be problematic. First of all, more observations are expected from areas with dense human population (higher possibility of observation due to higher research effort). Secondly, in many occasions there is a difficulty in determining species due to a lack of photographs showing the characteristic traits well. Third, some orthopteran species (e.g., *Meconemathalassinum* and *Barbitistesconstrictus*) live a hidden lifestyle and are less expected to be observed without particular searching. Some species are “not interesting” to a non-professional observer, due to their unremarkable appearance (e.g., *Chorthippus* spp.), while some are targeted by observers due to conservation status, interesting biology, or appearance (e.g., Oedipodidae species). This results in some species being underrepresented and others overrepresented. Even so, CS platforms are valuable tools to the scientific community, as they help to build knowledge.

Overall, we can expect additions to the local fauna in the coming years. As there is no monitoring programme for Orthoptera in Latvia, the distribution and population trends in Latvia are little known. However, such information on diversity is crucial to conservation biology.

## ﻿Conclusions

There are 43 species of Orthoptera in Latvia. Many of these species need more detailed information on occurrence, distribution and ecology, which could be achieved by a dedicated monitoring programme. A re-evaluation of the conservation status for multiple species is needed, especially those in the Oedipodinae subfamily.

## Supplementary materials

Supplementary material 1 Orthoptera occurrence data Data type occurrences Explanation note The data table contains over 1500 observations (dating from 2003 to 2021) of 42 Orthoptera species in Latvia. The columns contain the following information: A – species scientific name, B – observation date, C – data source, D and E – x and y coordinates in LKS-92 coordinate system, F and G – international coordinates (latitude, longitude). https://binary.pensoft.net/file/777903This dataset is made available under the Open Database License (http://opendatacommons.org/licenses/odbl/1.0/). The Open Database License (ODbL) is a license agreement intended to allow users to freely share, modify, and use this Dataset while maintaining this same freedom for others, provided that the original source and author(s) are credited.Rūta Starka, Uģis Piterāns, Voldemārs Spuņģis

Supplementary material 2 Observational notes on Orthoptera species in Latvia Data type text Explanation note Information from historic resources, ecology research, species lists and observation data bases is combined to give a brief analysis of the known history, habitat preferences and occurrence of Orthoptera species in Latvia is presented. All historically used synonyms for every species is provided. https://binary.pensoft.net/file/777904This dataset is made available under the Open Database License (http://opendatacommons.org/licenses/odbl/1.0/). The Open Database License (ODbL) is a license agreement intended to allow users to freely share, modify, and use this Dataset while maintaining this same freedom for others, provided that the original source and author(s) are credited.Rūta Starka, Uģis Piterāns, Voldemārs Spuņģis
