# Supplementary material for: ﻿Annotated catalogue of Orthoptera (Orthoptera, Insecta) of Latvia
Source: Zookeys. 2022 Dec 5;1134:39–52. doi: 10.3897/zookeys.1134.95637 (PMC9836421; doi:10.3897/zookeys.1134.95637)
Supplement: Supplementary material 2 — Observational notes on Orthoptera species in Latvia [file zookeys-1134-039_article-95637__-s002.pdf]

Suppelementary material 2

Observational notes on Orthoptera species in Latvia

Information from historic resources, ecology research, species lists and observation data bases is combined to give a brief analysis of the known history, habitat preferences and occurence of Orthoptera species in Latvia is presented. All historically used synonyms for every species is provided.

Suborder Caelifera

Family Acrididae MacLeay, 1821

Subfamily Calliptaminae Jacobson, 1905

(–) ***Calliptamus italicus*** (Linnaeus, 1758)

Synonyms used. *Caloptenus italicus* in Kawall (1864), *Calliptamus (Caloptenus) italicus* in Princis (1931, 1932).

Observational notes. The first and last records of *C. italicus* in Latvia dates back to 19th century (Kawall 1864), although the presence of this species was doubted shortly after (Princis 1943). The inclusion of this species in Latvia is based on one female specimen with a missing label in the historical entomological collection of B. A. Gimmerthal (Princis 1934a), and according to Kawall (1864), the specimen was supposedly collected in Livonia, a historical territory beyond the contemporary borders of Latvia. However, it is known, that B. A. Gimmerthal did most of his collecting in Rīga surroundings (Flor 1860). Nowadays, the closest known location from Latvia for this species is south of Belarus (California Academy of Sciences 2021). While in Fauna Europea (Willemse and Heller 2013) this species is marked as "doubtfully present", in a checklist of European Orthoptera fauna (Heller et al. 1998) this species is marked as absent from Latvia. We also conclude, that until an individual has been caught, we can assume this species has not been found in Latvia.

Subfamily Gomphocerinae Fieber, 1853

Chorthippus (Chorthippus) albomarginatus (De Geer, 1773)

Synonyms used. *Stenobothrus elegans* in Kawall (1864), *Chorthippus elegans* in Princis (1931, 1932, 1934b) and Spuris (1957).

Observational notes. This species is first recorded in Latvia by Kawall (1864). Some individuals are also preserved in Gimmerthal's collection (Princis 1934a). After that, numerous localities have been discovered (Princis 1943). Historically known form wet meadows (Spuris 1957). Nowadays it is a common species with high occurrence in cultivated grasslands, together with *Chorthippus dorsatus* (Rozenfelde et al. 2017), but can also be found in other grassland habitats (Matisons 2005).

Chorthippus (Chorthippus) dorsatus (Zetterstedt, 1821)

Synonyms used. *Stenobothrus dorsatus* in Kawall (1864).

Observational notes. Although Kawall mentioned this species as possible member of the local fauna, this common species was first recorded in Latvia only in 1932 (Princis 1933). After that, numerous

other localities were found across the country (Princis 1943). Today, very common in cultivated grasslands, often together with *C. albomarginatus* (Rozenfelde et al. 2017), and can also be found in other grassland habitats (Matisons 2005) and calcareous fens (Spuņģis 2013).

Chorthippus (Glyptobothrus) apricarius (Linnaeus, 1758)

Synonyms used. *Stauroderus apricarius* in Princis (1931, 1932) and Spuris (1957).

Observational notes. Historically known form dry, grassy areas (Spuris 1957). Results from recent studies point to disturbed habitats – grazed meadows and set asides (Matisons 2005, Rozenfelde et al. 2017), dry heathlands (Rozenfelde 2018). In these researches *C. apricarius* is present with a few individuals in each case. Also, only one observation can be gathered from CS platforms (California Academy of Sciences 2021). Therefore, data suggests that *C. apricarius* is not a very common species.

Chorthippus (Glyptobothrus) biguttulus (Linnaeus, 1758)

Synonyms used. *Stenobothrus variabilis* var. *biguttulus* in Kawall (1864), *Stauroderus biguttulus* in Princis (1931, 1932, 1934b) and Spuris (1957).

Observational notes. Known in Latvia since mid-19th century (Kawall 1864). Occurs in dry habitats, forest clearings (Spuris 1957). Common species in dry grasslands (Rozenfelde et al. 2017) and dry heathlands (Rozenfelde 2018). Rarely found in calcareous fens (Spuņģis 2013).

Chorthippus (Glyptobothrus) brunneus (Thunberg, 1815)

Synonyms used. *Stauroderus bicolor* in Princis (1931, 1932) and Spuris (1957), *Chorthippus bicolor* in Princis (1943).

Observational notes. This species has been very common throughout the history, with numerous known localities (Princis 1943). Occurs in grassland habitats (Rozenfelde et al. 2017), dry heathlands (Rozenfelde 2018), costal dunes (Spuņģis 2007), other dry habitats (Spuris 1957), road sides, forest clearings, forest edges and other grassy habitats (Matisons 2005). The species is not often recorded in CS platforms (California Academy of Sciences 2021, Latvijas Dabas Fonds and Latvijas Ornitoloģijas biedrība 2021). Nontheless, *C. brunneus* is very common (probably the most common Orthoptera species in Latvia) and distributed throughout the country.

(?) ***Chorthippus (Glyptobothrus) mollis*** (Charpentier, 1825)

Synonyms used. *Stauroderus mollis* in Princis (1931).

Observational notes. Unclear distribution and occurrence in Latvia. Supposedly found in dry places (Spuris 1957), but the last and only known record is from 1931 (obs. K. Princis) from Priedaine. The species is marked "present" in Latvia and the neighbouring countries (Willemse and Heller 2013), and some of the localities are fairly close to Latvian border (Albrecht 1963, Budrys and Pakalniškis 2007, California Academy of Sciences 2021). But for now, there are no individuals preserved in entomological collections, nor are there any observations registered in CS platforms.

Chorthippus (Glyptobothrus) pullus (Philippi, 1830)

Synonyms used. *Stauroderus pullus* in Princis (1931, 1934b, 1935).

Observational notes. This species was first observed in Latvia in 1933 (Princis 1934b). In the European Orthoptera species list (Heller et al. 1998) it is marked as present in Baltics. The species was recently recorded in calcareous grasslands in Abava river valley (Rozenfelde et al. 2017). But since there are no other information about this species occurrence in Latvia, and the recent information in Europe is scarce (California Academy of Sciences 2021, Willemse and Heller 2013), distribution needs further investigation.

Chorthippus (Glyptobothrus) vagans (Eversmann, 1848)

Observational notes. This species is not mentioned in the historical literature, therefore also marked as absent in Fauna Europea (Willemse and Heller 2013). This species occurs in Latvia in dry meadows and grasslands (Matisons 2005, Rozenfelde et al. 2017), costal dunes (Spuņģis 2007) and dry heathlands (Rozenfelde 2018) (Fig. 1a). This is the first time the species is added to the check-list of local fauna.

Chrysochraon dispar (Germar, 1834).

Observational notes. The species was first predicted to be found in Latvia by Kawall (1864). In 1932, Princis has found it in Ovīši, and later in numerous other sites across the country (Princis 1932, 1943). This species is fairly common in fens (Spuņģis 2013) and wet meadows throughout the country (Latvijas Dabas Fonds and Latvijas Ornitoloģijas biedrība 2021).

Euthystira brachyptera (Ocskay, 1826).

Synonyms used. *Chrysochraon brachypterus* in Princis (1931, 1934b).

Observational notes. This species is known in Latvia since 1933, when it was found in Ogre (Princis 1934b). At the time *E. brachyptera* was considered to be quite rare (Princis 1943). Although the species is marked "absent" from Latvia in Fauna Europaea (Willemse and Heller 2013), and there are no observations registered in "iNaturalist" (California Academy of Sciences 2021), *E. brachyptera* is common in calcareous grasslands (Rozenfelde et al. 2017) and calcareous fens (Spuņģis 2013), as well other grassland habitats and dry road sides (Latvijas Dabas Fonds and Latvijas Ornitoloģijas biedrība 2021).

(?) ***Gomphocerippus rufus*** (Linnaeus, 1758)

Synonyms used. *Gryllus rufus* in Fischer (1778), *Stenobothrus rufus* in Kawall (1864), *Gomphocerus rufus* in Princis (1931, 1932, 1943).

Observational notes. Historical literature point to doubtful occurrence in Latvia. This species was first marked by Fischer (1778), mentioning meadows as this species habitat. Kawall (1864) also lists the species, but later reviews of Kawall's work indicate an error – no individuals were found in his entomological collection (Princis 1943). As the species is found in the surrounding countries, Princis considered this species to be potentially present in Latvia (Princis 1931, 1943). In Lithuania, this species also has no documented findings, but it is listed as present due to overall distribution area of this species (Budrys and Pakalniškis 2007). In Estonia, there is one observation from 1931 (Albrecht 1963). In Latvia, the species is marked "present" in Fauna Europea (Willemse and Heller 2013), but the information source could be doubted – there are no preserved specimens in collections, nor any other types of records of this species in Latvia to this day. The closest recent observation to Latvia is

in Belarus (California Academy of Sciences 2021). Therefore, the presence of this species in Latvia remains unproven and doubtful.

Myrmeleotettix maculatus (Thunberg, 1815)

Synonyms used. *Stenobothrus biguttatus* in Kawall (1864), *Gomphocerus maculatus* in Princis (1931, 1932) and Spuris (1957), *Gomphocerus biguttatus* in Princis (1934a), *Myrmeleotettix (Gomphocerus) maculatus* in Princis (1934b).

Observational notes. Known in Latvia since mid-19th century (Kawall 1864). Common species in costal dunes (Spuņģis 2007), dry heathlands (Rozenfelde 2018), forest clearings and other very dry, xerophytic habitats (California Academy of Sciences 2021; Latvijas Dabas Fonds and Latvijas Ornitoloģijas biedrība 2021; Spuris 1957).

Omocestus (Omocestus) haemorrhoidalis (Charpentier, 1825)

Observational notes. This species was first observed in Latvia in 1933 (Princis 1934b). Later, a few more localities were described, mostly from the central part of the country (Princis 1943). This species has been recorded from dry heathlands (Rozenfelde 2018), calcareous grasslands (Rozenfelde et al. 2017), set-aside meadows (Matisons 2005) and coastal dunes (Spuņģis 2007). Not very common.

(?) ***Omocestus (Omocestus) rufipes*** (Zetterstedt, 1821)

Synonyms used. *Stenobothrus rufipes* in Kawall (1864), *Omocestus ventralis* in Matisons (2005).

Observational notes. Historically, this species has been known from Courland, western Latvia (Kawall 1864; Princis 1931), but not much details are known about the localities. Later, an individual has been collected in Dobele, in a sports field next to castle ruins (Princis 1939). In Latvian key to Orthoptera, it is mentioned that the species habitat is dry meadows and forest clearings (Spuris 1957), but there is no additional information on what observations this habitat has been derived (possibly from foreign literature). In Fauna Europea the species is marked as present in Latvia (Willemse and Heller 2013), but again the information source is unknown. In Lithuania and Estonia, however, the species status is "doubtfully present" (Willemse and Heller 2013). There is one local grassland study, where it has been found in set-aside meadows, cultivated grasslands and *Sesleria caerulea* grassland (Matisons 2005). As there are no individuals preserved in entomological collections available for verification, it is possible, that the species was misidentified. There are no observations of this species in Lithuania (Budrys and Pakalniškis 2007), nor in Latvia in any CS platform. Therefore, it necessary to investigate the historical location and the surrounding area for more proof.

Omocestus (Omocestus) viridulus (Linnaeus, 1758)

Synonyms used. *Gryllus viridulus* in Fischer (1778), *Stenobothrus viridulus* in Kawall (1864), *Gomphocerus viridulus* in Princis (1934a).

Observational notes. This is one of the earliest known species in Latvia. Fischer (1778) describes, that *O. viridulus* can be found in meadows. Historically it was known to be a common species across the country (Princis 1943) and that is true also for today (probably one of the most common Acrididae species in Latvia). It can be found in a variety of habitats along the humidity gradient – dry heathlands (Rozenfelde 2018), grasslands (Matisons 2005; Rozenfelde et al. 2017), calcareous fens (authors' observations) and other habitats (Latvijas Dabas Fonds and Latvijas Ornitoloģijas biedrība 2021).

Pseudochorthippus montanus (Charpentier, 1825)

Synonyms used. *Chorthippus longicornis* in Princis (1931, 1932, 1933, 1943) and Spuris (1957), *Stauroderus (Chorthippus) longicornis* in Princis (1934b).

Observational notes. The species was first found by Princis in Ovīši and Jēkabpils surroundings (Princis 1931), but later from numerous other localities (Princis 1943). Historically known from wet meadows (Princis 1931; Spuris 1957). Macropterous form has also been recorded (Princis 1934b). Nowadays it is common species in fen habitats (Spuņģis 2013), and fairly common in wet grasslands.

Pseudochorthippus parallelus (Zetterstedt, 1821)

Synonyms used. *Stenobothrus pratorum* in Kawall (1864), *Chorthippus parallelus* in Princis (1931, 1932, 1943) and Spuris (1957), *Stauroderus parallelus* in Princis (1934b), *Stauroderus (Chorthippus) parallelus* in Princis (1935).

Observational notes. Known in Latvia since mid-19th century (Kawall 1864). Historically found across the country (Princis 1943) in humid grasslands (Spuris 1957). Today, a very common species in variety of grassland habitats – seminatural calcareous grasslands, as well as cultivated and ruderal grasslands (Rozenfelde et al. 2017). A macropterous individual (*Pseudochorthippus parallelus* f. *explicatus*) has been recorded in Abava river valley (Rozenfelde 2017).

Stauroderus scalaris (Fischer von Waldheim, 1846)

Synonyms used. *Stauroderus morio* in Princis (1931).

Observational notes. In 1931 Princis discussed the possibility of finding *S. scalaris* in Latvia (Princis 1931). Later he was still reluctant to include this species a permanent member of local fauna (Princis 1943), as there was just one misidentified individual of *S. scalaris* in Gimmerthal's collection from Livonia (unspecified location), and at the time it was the only known documentation of this species in Latvia (Princis 1934a, 1943). Nowadays, *S. scalaris* has been found in dry heathlands (Rozenfelde 2018) and several grassland habitats (Matisons 2005; Rozenfelde et al. 2017). However, Latvia seem to be on the northern border of the species distribution area (California Academy of Sciences 2021; Willemse and Heller 2013).

Stenobothrus lineatus (Panzer, 1796)

Synonyms used. *Gomphocerus lineatus* in Princis (1934a).

Observational notes. First recorded in Latvia by Kawall (1864). Later, several other localities across the country were discovered (Princis 1943). Two individuals from Sērene and Tome (leg. Princis) are stored in the LMNH entomological collection. The species is known from dry, sunny places (Spuris 1957). Recently, two individuals were recorded in dry, calcareous grasslands in Abava river valley, where horse grazing was used as a management practice (Rozenfelde 2017). No other information is available on this species in Latvia, including all CS platforms, therefore it is possibly rather rare.

Stenobothrus stigmaticus (Rambur, 1838)

Observational notes. First recorded in Latvia by Princis, and immediately with numerous localities (Princis 1932, 1934b, 1943). The species was known from dry, grassy areas (Spuris 1957). In recent

research it has been found in grazed, dry, calcareous grasslands (together with *S. lineatus*), as well as in calcareous grasslands with no management (Rozenfelde 2017), and calcareous fens (authors' observations). This species is uncommon.

Subfamily Melanoplinae Scudder, 1897

Podisma pedestris (Linnaeus, 1758)

Synonyms used. *Pezotettix pedestris* in Kawall (1864), *Gomphocerus pedestris* in Princis (1934a).

Observational notes. First recorded in Latvia by Kawall (1864), but a couple of individuals were also preserved in Gimmerthal's collection (Princis 1934a). This is a species with declining occurrence – in 1931 Princis mentions that this species is not very common, while nowadays we can say that it is definitely a rare species, with few recent records. *P. pedestris* can be found in dry habitats (road sides, forest clearings, forest edges, clear-cuts), and is distributed mostly in Rīga surroundings (Ādaži, Garkalne, Ropaži) with few exceptions (Latvijas Dabas Fonds and Latvijas Ornitoloģijas biedrība 2021, authors' observations). The limited number of observations could also be due to low dispersal abilities and environment-adapted appearance of this species. *Podisma pedestris* is included in the protected species list in Latvia (Ministru kabinets 2004b).

Subfamily Oedipodinae Walker, 1871

(?) ***Bryodemella (Bryodemella) tuberculata*** (Fabricius, 1775)

Synonyms used. *Oedipoda tuberculata* in Kawall (1864), *Bryodema tuberculata* in Princis (1931, 1939, 1943), *Bryodema tuberculatum* in Z. Spuris (1998).

Observational notes. The species was first predicted to be found in Latvia by Kawall (1864). Then, Princis (1939) has identified some individuals of this species in 1924 (from Garciems), 1928 (from Sauka) and 1936 (from Gulbene). The individual from Sauka is currently still in the LMNH entomological collection in Rīga. Since then, no other individuals have been recorded in Latvia, and it is considered locally extinct (Spuris 1998; Zuna-Kratky et al., 2016), even if in some regional species lists it is marked as present (Heller et al. 1998; Willemse and Heller 2013). At the moment, the closest known locality for this species is in a military area in southern part of Lithuania (Ūsaitis, 2019).

Locusta migratoria (Linnaeus, 1758)

Synonyms used. *Gryllus migratorius* in Fischer (1778), *Pachytilus migratorius* in Kawall (1864).

Observational notes. This species is first documented in Latvia in the 15th century, when apparently a swarm had reached Daugavpils surroundings and had distributed further along the Daugava river (Ozols 1963; Princis 1943). Princis (1943) has described the history of this species in Latvia in length. He and other authors (Fischer 1778; Kawall 1864; Ozols 1963) point out, that there are no established populations in Latvia. Mostly solitary individuals, or small groups of individuals can be found in years, when swarming has been observed in the nearby southern populations (Ozols 1963). Most of the historic localities compiled by Princis are around Rīga (Princis 1943). Recent observations are also from the Gulf of Riga costal dunes (Rīga, Mērsrags) and in inland – Suntaži (Ogre municipality) (Latvijas Dabas Fonds and Latvijas Ornitoloģijas biedrība 2021). Nymphal stages have never been found in Latvia, confirming that this species is only a rare accidental visitor.

Oedipoda caerulescens (Linnaeus, 1758)

Synonyms used. *Oedipoda coerulescens* in Princis (1943).

Observational notes. The review of Princis points to 1917 as the first time the species has been mentioned in Latvia (Zacher 1917, cited from Princis 1943), although the information source is list of Orthoptera in Germany. One female individual has been preserved in Gimmerthal's collection (Princis 1934a), which is probably the first reliable record. Later Princis mentions a few other localities where he has found the species (Princis 1943). *O. caerulescens* was thought to be very rare (Princis 1931, 1932), and was known from dry, sandy places with sparse vegetation, such as dunes, heathlands and similar habitats (Spuris 1957, 1998). Together with *Sphingonotus caerulans*, *O. caerulescens* was included in the 1st category in the Red Data Book of rare and endangered species in Latvia (Spuris 1998), and later also in the list of endangered species in Latvia (Ministru kabinets 2004b). In 2013 Latvian Entomological society nominated *O. careulescens* as "Insect of the Year", after which numerous new localities were discovered. Observations of *O. caerulescens* are numerous nowadays (Latvijas Dabas Fonds and Latvijas Ornitoloģijas biedrība 2021). It is commonly found in dry heathlands (Rozenfelde 2018), coastal dunes (Spuņģis 2007), forest clearings, quarries and other dry, sandy habitats.

(–) ***Oedipoda miniata*** (Pallas, 1771)

Observational notes. A label with the name of this species was found in the historical collection of B. A. Gimmerthal, but without any individual to support the legitimacy of the find (Princis 1934a). Later Princis discusses, weather the species in question wasn't in fact *Oedipoda germanica* (Princis 1943). Princis doubted the possibility to find either of these southern species in Latvia, especially because there was no preserved individual, and the whole discussion of *O. miniata* and *O. germanica* in Latvia was based on what possibly was a misassumption of a single label with no individual in Gimmerthal's collection (Princis 1943). Later, the name of this species appears in an unofficial online species list (Matisons 2004), but probably also due to misinterpretation of the historic records. This species clearly has a southern distribution (California Academy of Sciences 2021; Willemse and Heller 2013), and therefore it is very unlikely to have occurred in Latvia.

Psophus stridulus (Linnaeus, 1758)

Synonyms used. *Gryllus stridulus* in Fischer (1778), *Pachytilus stridulus* in Kawall (1864).

Observational notes. This species is known in Latvia since 18th century (Fischer 1778). Princis (1931) describes this species as fairly common throughout the country, but nowadays, the species distribution seems to be limited to coastal areas (the Maritime lowland) and is rare further inlands (Latvijas Dabas Fonds and Latvijas Ornitoloģijas biedrība 2021). This species is found only in suitable habitats – costal dunes, heathlands, dry meadows, south facing slopes. It has been recorded that the species prefers dry heathlands where controlled burning is used as habitat management, rather than overgrown heaths (Rozenfelde 2018).

Sphingonotus (Sphingonotus) caerulans (Linnaeus, 1767)

Synonyms used. *Oedipoda cyanoptera* in Kawall (1864), *Sphingonotus coerulans cyanopterus* in Princis (1931, 1936, 1943), *Sphyngonotus cyanopterus* in Princis (1932).

Observational notes. This species is known in Latvia since the 19th century (Kawall 1864), but has always remained rare. Princis mentions some findings near Rīga and a few other locations, all situated in coastal area (Princis 1931, 1936, 1943). There are five individuals preserved in the LMNH entomological collection – from Rīga, 1937 (Leg. J.Muskars), Vangaži, 1950 (Leg. O. Ceriņš), Mangaļi, 1956 (Leg. G.Ozols), Carņikava, 1978 (Leg. L.Danka) and from Garupe, 1979 (Leg. M. Stiprais). The species has been included in the 1st category in the Red Data Book of rare and endangered species in Latvia (Spuris 1998), and later for a short time (from 2000 to 2004) in list of protected species (Ministru kabinets 2004b, 2004a). Last observations of this species are from 2013 (Gulbene municipality), 2014 (Smiltene municipality) and 2015 (Balvi and Gulbene municipalities) (M. Kalniņš unpublished data). After checking two of the recent localities in 2021, the habitat was overgrown and the species was not found (authors' observation). Regardless of no legal conservation status, this species is extremely rare in Latvia, more so than *O. coerulescens*, and research on distribution, as well as re-establishing the conservation status is strongly needed.

Stethophyma grossum (Linnaeus, 1758)

Synonyms used. *Mecostethus grossus* in Princis (1931, 1932, 1934a, 1934b, 1943) and Spuris (1957), *Gomphocerus grossus* in Princis (1934a).

Observational notes. This species is known in Latvia since mid-19th century (Kawall 1864), mostly from the west side of the country (Princis 1943). Some individuals are also preserved in Gimmerthal's collection (Princis 1934a). It was known to be fairly common in wet grasslands (Princis 1931; Spuris 1957). Today, the species has also been found in other humid habitats like raised bogs, calcareous fens (Spuņģis 2013). Although the species is mentioned as "least concern" in IUCN red-list globally, multiple European countries have listed *S. grossum* as vulnerable or near threatened, and conservation actions are taking place (Hochkirch et al. 2016f). There are multiple observations registered in CS platforms, mostly from western and central part of the country (California Academy of Sciences 2021; Latvijas Dabas Fonds and Latvijas Ornitoloģijas biedrība 2021).

Family Tetrigidae Rambur, 1838

Tetrix bipunctata (Linnaeus, 1758)

Synonyms used. *Acrydium bipunctatum* in Princis (1931, 1932, 1933, 1934b) and Spuris (1957), *Tetrix bipunctatum* in Spuņģis and Kalniņš (2002).

Observational notes. Known in Latvia since mid-19th century (Kawall 1864), and since then, it has been considered a common species (Kawall 1864; Spuris 1957). Princis points out, that Kawall's conclusion of the species occurrence has to be taken with caution, because at that time *T. undulata* and *T. tenuicornis* were also determined as *T. bipunctata* and the definitive delimitation of these 3 species from one another was not made until later (Princis 1943). The species has been found in dry heathlands (Rozenfelde 2018), calcareous grasslands (Rozenfelde et al. 2017), as well as calcareous fens (Spuņģis 2013). There are not many observations in CS platforms, but the species seems to be distributed mostly along the coastline (Latvijas Dabas Fonds and Latvijas Ornitoloģijas biedrība 2021).

(–) ***Tetrix kraussi*** Saulcy, 1888

Synonyms used. *Acrydium kraussi* in Princis (1931, 1932, 1934a, 1934b).

Observational notes. Princis predicted this species in his publication in 1931, and one year later he describes his finding of some male and female specimens from Dubulti and Ovīši (Princis 1932) and later already from 5 different localities (Princis 1934b). From some of his work (Princis 1934a, 1936) it can be understood, that back then *T. kraussi* was considered a synonym of *T. bipunctata*, so there are some problems in interpreting his work when it comes to this genus. No other records of *T. kraussi* have been registered since then, and the species distribution area is confined to Central and Southern Europe (California Academy of Sciences 2021; Cigliano et al. 2021). Therefore, we conclude that this species has not been recorded in Latvia, and all previous records should be attributed to *T. bipunctata*.

Tetrix subulata (Linnaeus, 1758)

Synonyms used. *Acrydium subulatum* in Princis (1931, 1932, 1933, 1934a, 1943) and Spuris (1957).

Observational notes. The first time *T. subulata* is recorded in Latvia is in mid-19th century (Kawall 1864). A couple of individuals had been preserved in Gimmerthal's collection (Princis 1934a). Princis has observed this species many times in numerous locations across the country (Princis 1943), and historically it has always been considered to be a common species (Kawall 1864; Spuris 1957). *T. subulata* can be found in both dry habitats, such as heathlands (Rozenfelde 2018) and coastal dunes (Spuņģis 2007), as well as in more humid habitats, such as calcareous fens (Spuņģis 2013), and it is a quite common species (Latvijas Dabas Fonds and Latvijas Ornitoloģijas biedrība 2021).

Tetrix tenuicornis (Sahlberg, 1891)

Synonyms used. *Acrydium tenuicornis* in Princis (1934a), *Acrydium tenuicorne* in Princis (1943).

Observational notes. The first recorded individuals of this species were found in Gimmerthal's collection (Princis 1934a). Since then, a few more localities have been mentioned by Princis (1943), mostly from the East side of the country. Several individuals from Koknese, 1943 (Leg. L. Danka and E. Strands) are stored in LMNH entomological collection. There are two recent observations. First, from a set-aside meadow in Ķemeri National park (Matisons 2005), but the individual is not preserved, therefore the record is unsure. Second observation is from Ogre, 2010, when an individual was photographed on the railway (Latvijas Dabas Fonds and Latvijas Ornitoloģijas biedrība 2021). The lack of knowledge on this species could be due to the miniature size and complicated species determination. Considering the vast distribution area (Willemse and Heller 2013), this species is probably more common then it appears.

Tetrix undulata (Sowerby, 1806)

Synonyms used. *Acrydium kiefferi* in Princis (1931, 1933, 1934b, 1943).

Observational notes. Princis first mentioned this species as potential for Latvia in 1931. Later in 1933 he had found some individuals in Dubulti and Ķemeri. He described the species as quite common (Princis 1931, 1933). He also mentioned finding macropterous form in Ķemeri (Princis 1932). Later he described the species distribution in Latvia to be situated west from the Daugava river (Princis 1943). Although there are no records in CS platforms, considering the distribution area (Willemse and Heller 2013), it is probably a common species. It has been recorded in dry heathlands in two subsequent years (Rozenfelde 2018), and also in a calcareous fen in Platene (authors' unpublished data, pitfall traps, 2018). Habitats with bare soil and high humidity are preferred by this species.

Suborder Ensifera

Family Gryllidae Laicharting, 1781

Acheta domesticus (Linnaeus, 1758)

Synonyms used. *Gryllus domesticus* in Fischer (1778), Kawall (1864), Princis (1931, 1932, 1934a) and Spuris (1957), *Gryllulus domesticus* in Princis (1943).

Observational notes. Notes on this species in Latvia first appear soon after its description (Fischer 1778), when it is referred to as "well known". It is also listed in the fauna of Latvia later by Kawall (1864) and Princis (1931, 1943). According to Princis (1931), the species occurred throughout the country, but after this work, there was a longer period with no observations. This synanthropic species was described to inhabit countryside house kitchens and bread bakeries (Spuris 1957). After the Entomological Society of Latvia nominated *A. domesticus* as the Insect of the Year 2002, new information on the species distribution was obtained. The species is since then known to be mainly distributed in central and western part of Latvia (Gailis et al. 2003), but more recent observations are also from eastern parts of the country (Latvijas Dabas Fonds and Latvijas Ornitoloģijas biedrība 2021). Rather rare.

(–) ***Gryllus (Gryllus) campestris*** Linnaeus, 1758

Synonyms used. *Liogryllus campestris* in Princis (1931, 1932, 1934a) and Spuris (1957).

Observational notes. First records date back to 18th century (Fischer 1778), but no detailed information on occurrence or distribution is provided. Later, Kawall (1864) considered *G. campestris* to be an alien species, because after searching for it in possible locations in Courland, he had never found it himself. In the mid-20th century the species presence in Latvia was also doubted (Princis 1931, 1943), yet not denied (Princis 1932). According to Princis, the only known specimen from former Livonia territory was to be found in B. A. Gimmerthal's entomological collection, one well preserved female (Princis 1934a). There are still no findings of this species in Latvia since then, and it can be assumed that field cricket is not present in the local fauna. Latvia is also on the north border of this species distribution area, and the closest known location is in south of Lithuania (California Academy of Sciences 2021).

Family Gryllotalpidae Leach, 1815

Gryllotalpa gryllotalpa (Linnaeus, 1758)

Synonyms used. *Gryllus gryllotalpa* in Fischer (1778), *Gryllotalpa vulgaris* in Kawall (1864) and Princis (1931, 1932, 1934a).

Observational notes. This species is also known from Latvia since the earliest faunal list. Then, it is noted that *G. gryllotalpa* is fairly common around Rīga, but with low population density (Fischer 1778). Later more localities in wider geographical range were described (Princis 1931, 1943). It has been considered to be agricultural pest species (Ozols 1963), and is known to prefer humid soils (Spuris 1957). After nominating this species as Insect of the Year 2007, the knowledge on distribution increased slightly. Today, the species is found in all regions in Latvia, but not equally. Most localities are still recorded from Rīga surroundings and the Maritime lowland (California Academy of Sciences 2021; Latvijas Dabas Fonds and Latvijas Ornitoloģijas biedrība 2021). It seems that the distribution and occurrence of mole cricket has remained stable throughout the time.

Family Rhaphidophoridae Walker, 1869

Tachycines (Tachycines) asynamorus Adelung, 1902

Observational notes. First records are from greenhouse of the Botanical Garden of University of Latvia (Princis 1932). It maintained a stable population in these greenhouses in the subsequent years (Princis, 1936), but there are no data if the species has survived in the same location until present time. A new locality for this species was recorded in 2001 in Daugavpils, when a quite large population was found in the city's heating network boiler room (Gailis et al. 2003). No other records of this species exist, but it is clear that the species is introduced and capable of survival only in synanthropic conditions.

Family Tettigoniidae Krauss, 1902

Barbitistes constrictus Brunner von Wattenwyl, 1878

Observational notes. This species has been first recorded in Latvia in 1937 near Rīga, and at the time, it was also the northernmost find for this species overall distribution area (Princis 1939). Then it was discussed, whether this species has a potential of becoming a forest pest species, but these predictions have not come true. After that few other historical records were reported (Princis 1943). Recently numerous adult and nymph observations have been reported from new localities between 2005 – 2021, mostly in coastal forests, but also in inland areas (Latvijas Dabas Fonds and Latvijas Ornitoloģijas biedrība 2021). As this species is difficult to observe, it is hard to evaluate the occurrence.

Conocephalus (Anisoptera) dorsalis (Latreille, 1804)

Synonyms used. *Xiphidium dorsale* in Kawall (1864), *Conocephalus (Xiphidium) dorsalis* in Princis (1932), *Conocephalus dorsalis* in Princis (1943).

Observational notes. This species was first recorded in Latvia in 1931 (Princis 1931), but before that, Kawall (1864) mentioned this species as possibly found in Latvia. Later only a few new localities were found (Princis 1943). It was known from humid and swampy places (Spuris 1957). Today, this species is common, but restricted to suitable habitats, such as damp meadows and fens. As fen habitats in Latvia are distributed mostly along Baltic sea coastline, the recorded observations of this species between 2013 – 2019 are also mostly from coastal areas (California Academy of Sciences 2021; Latvijas Dabas Fonds and Latvijas Ornitoloģijas biedrība 2021).

Conocephalus (Anisoptera) fuscus (Fabricius, 1793)

Synonyms used. *Xiphidium fuscum* in Kawall (1864).

Observational notes. Very rare in Latvia, with only two observations (on a forest edge between Daugavpils and Aglona in 2012, and in a forest clearing near Bauska in 2020), both in the south of Latvia (Fig. 1b). This is not surprising, as Latvia is on the northern border of the species distribution area (Willemse and Heller 2013). Historically, Kawall (1864) only mentions this species as potentially found in Latvia. There is no other information on the occurrence of this species, and this is the first time it is added to the check-list of Latvian Orthoptera species.

Decticus verrucivorus (Linnaeus, 1758)

Synonyms used. *Gryllus verrucivorus* in Fischer (1778).

Observational notes. This species is known to be common in grasslands since the earliest known records (Fischer 1778; Kawall 1864; Princis 1943; Spuris 1957). In local research it has been recorded from dry heathlands (Rozenfelde 2018), dry, calcareous grasslands (Rozenfelde 2017) as well as other grassland habitats (Matisons 2005) and costal dunes (Spuņģis 2007). In Latvia, the species is considered characteristic species for natural grassland habitats – xeric and calcareous grasslands, semi-natural dry grasslands and scrubland facies on calcareous substrates, and species-rich *Nardus* grasslands on siliceous substrates in mountain areas (Rūsiņa 2013). Common throughout the country (California Academy of Sciences 2021; Latvijas Dabas Fonds and Latvijas Ornitoloģijas biedrība 2021).

Leptophyes punctatissima (Bosc, 1792)

Synonyms used. *Odontara punctatissima* in Kawall (1864), *Leptophyes punctatissema* in Princis (1931).

Observational notes. This species has been mentioned by Kawall (1864) and Princis (1931) as a potential species in Latvia. Only now, some 150 years after Kawall's prediction, we can report on the first observations. A single female individual was observed and collected on August, 2020 in Garupe, Carņikava municipality, sitting on an old orange tree in a garden (Leg. Z. Zamuška, Det. U. Piterāns, R. Rozenfelde). This finding, however, is confusing, as we can't exclude the possibility that the individual was imported with some garden plants as the surrounding territory is developing with new settlements. There are other findings of this species in 2021 – one from Rīga, but it is only documented with photographs, and the individual was not caught (Latvijas Dabas Fonds and Latvijas Ornitoloģijas biedrība 2021), and another one from Liepāja (California Academy of Sciences 2021) (Fig. 1c). As all current observations are in urban areas, the origins of observed specimens are unclear. Further studies are needed to clarify occurrence of this species in Latvia. This is a new species to Latvia.

Meconema thalassinum (De Geer, 1773)

Synonyms used. *Meconema varium* in Princis (1931).

Observational notes. Princis (1931) anticipated this species in Latvia, but later this species disappears from his works. There are five previously unpublished recent records of this species in Latvia. First record is from August 2008, when a female was trapped while climbing a stump of an oak tree in Rīga (Leg. M. Kalniņš). Later observations are mostly from Rīga surroundings – from Mārupe municipality, 2016 (Leg. R. Rozenfelde, a dead individual indoors), from a garden in Carņikava municipality, 2020 (Leg. N. Savenkovs, pheromone traps of *Synanthedon myopaeformis*), a garden in Jūrmala city, 2021 (registered in Dabasdati.lv, Det. U. Piterāns), and another dead individual indoors in Sigulda, 2021 (Leg. F. Šitca, Det. U. Piterāns) (Fig. 1d). The indoors observations of dead individuals might be due to the species attraction to light. Latvia is on the northern border of the species distribution area (Willemse and Heller 2013), so it is possible that the species arrived in Latvia in the recent years. Overall, *M. thalassinum* appears to be rare or difficult to observe due to nocturnal behaviour and no stridulation. This is another new species to Latvia.

Bicolorana bicolor (Philippi, 1830)

Synonyms used. *Metrioptera bicolor* in Princis (1931).

Observational notes. This species is another new addition to the Latvian Orthoptera check-list and at the same time the rarest species of the *Metrioptera* genus group locally. There are no historical records,

but Princis (1931) anticipated this species in Latvia. The first record of this species is from 2014, when it was found near the Belarussian border, and the second time in 2017 in Salaspils (Latvijas Dabas Fonds and Latvijas Ornitoloģijas biedrība 2021) (Fig. 1e). The occurrence of this species needs further investigation.

Metrioptera brachyptera (Linnaeus, 1761)

Synonyms used. *Platycleis brachypterus* in Kawall (1864), *Metrioptera (Platycleis) brachyptera* in Princis (1932).

Observational notes. This species has been known in Latvia since the 19th century, and already then it was noted as “not uncommon” (Kawall 1864). In the 20th century numerous localities were recorded (Princis 1943). Today this species in Latvia is very common in various habitats – grasslands, fens, road sides, heathlands (Latvijas Dabas Fonds and Latvijas Ornitoloģijas biedrība 2021). This species has a preference to slightly humid microhabitats (Rozenfelde et al. 2017). Macropterous form has also been observed.

Roeseliana roeselii (Hagenbach, 1822)

Synonyms used. *Decticus brevipennis* in Kawall (1864), *Metrioptera roeselii* in Princis (1931, 1934b).

Observational notes. The species was first predicted to be found in Latvia by Kawall (1864). This species has long been known to be common in Latvia (Princis 1931, 1943). The short-winged form (*M. roeselii* f. *diluta*) was recorded in 1933 (Princis 1934b). A macropterous individual has also been found in heathlands of Ādaži in 2012 (authors’ observation, pitfall traps). This species is nowadays the most commonly reported species of the *Metrioptera* genus group (California Academy of Sciences 2021; Latvijas Dabas Fonds and Latvijas Ornitoloģijas biedrība 2021), and can be the dominant Orthoptera species in overgrown heathlands (Rozenfelde 2018).

Phaneroptera (Phaneroptera) falcata (Poda, 1761)

Observational notes. This species was first discovered in Latvia in 2010 (Latvijas Dabas Fonds and Latvijas Ornitoloģijas biedrība 2021), but the official report was published two years later (Sokolovskis and Suveizda 2012). Today, it is clear that this species is a permanent member of the local fauna, as it can be found in most parts of the country (California Academy of Sciences 2021; Latvijas Dabas Fonds and Latvijas Ornitoloģijas biedrība 2021), and nymphal stages have also been observed. The species can be found in a variety of habitats – roadsides, gardens, grasslands, forest clearings, heathlands and fens.

Pholidoptera griseoaptera (De Geer 1773)

Synonyms used. *Thamnotrizon cinereus* in Kawall (1864), *Pholidoptera cinerea* in Princis (1931, 1934b), *Pholidoptera (Thamnotrizon) cinerea* in Princis (1932).

Observational notes. The species was first predicted to be found in Latvia by Kawall (1864). Years later, between 1931 and 1943, Princis reported 5 localities for this species (Princis 1943). The first place where the species was found was Ovīši (Princis 1931), where it can still be found today. The recent records from 2009 to 2020 suggest a coastal distribution (California Academy of Sciences 2021; Latvijas Dabas Fonds and Latvijas Ornitoloģijas biedrība 2021), similar to the one of *Conocephalus*

dorsalis. Some observations are also from the coast of Daugava and other rivers. This common species can be found in damp places like roadsides with diches and hygrophytic vegetation, fens, river banks and other humid habitats.

(?) ***Platycleis grisea*** (Fabricius, 1781)

(?) ***Platycleis albopunctata*** (Goeze, 1778)

Synonyms used. *Metrioptera grisea* in Princis (1931).

Observational notes. Princis (1931) anticipated *P. grisea* in Latvia due to its distribution in Europe and Russia. But only a single male specimen from one locality has been recorded by him in 1935 in Latvia, near lake Ummis, Carņikava municipality (Princis 1936, 1943). This individual was identified as *P. grisea* (Princis 1936, 1943), but this species nowadays has confusing taxonomy. In some sources it is treated as a separate species *P. grisea* (Cigliano et al. 2021; Massa and Fontana 2011), in others – as a subspecies *P. albopunctata grisea* (Sardet et al. 2021). It is highly possible, that in the historic find the individual is misidentified with *P. albopunctata*, since the two species can be easily confused (Heller et al. 1998; Massa and Fontana 2011; Sardet et al. 2021). As the individual has not been preserved in an entomological collection, there is no way to confirm or deny the validity of Princis identification. *Platycleis grisea* is also not officially listed in the neighbouring Lithuanian Orthoptera fauna, whereas *P. albopunctata* is common along the Baltic sea coastline (Budrys and Pakalniškis 2007). Overall, even if the species (as *P. albopunctata*) is marked as present in Latvia, Lithuania and Estonia (Willemse and Heller 2013), due to no other findings whatsoever, the presence of this species in Latvia is not confirmed.

Tettigonia cantans (Fuessly, 1775)

Synonyms used. *Locusta cantans* in Kawall (1864).

Observational notes. First recorded in Latvia in the mid-19th century (Kawall 1864), *T. cantans* has remained a common species throughout time (Heller et al. 1998; Princis 1932, 1943). The species distribution in the country is more or less even (California Academy of Sciences 2021; Latvijas Dabas Fonds and Latvijas Ornitoloģijas biedrība 2021), and it can be found in variety of habitats – forest edges and clearings, bushlands, gardens, road sides and other. Probably the most common Tettigoniidae species in Latvia.

Tettigonia viridissima (Linnaeus, 1758)

Synonyms used. *Gryllus viridissimus* in Fischer (1778), *Locusta viridissima* in Kawall (1864) and Princis (1934a), *Tettigonia (Locusta) viridissima* in Princis (1932).

Observational notes. *Tettigonia viridissima* was the first bush-cricket from *Tettigonia* genus to be mentioned in local fauna. It was known then to be rather uncommon, and found in woody areas and meadows (Fischer 1778). It was added later, that *T. viridissima* occurs more rarely than *T. cantans* (Kawall 1864), which is also true today. Princis (1943) also mentioned only a few localities in his faunal review (mostly coastal areas). Today the species is mostly known from central Latvia, with a few exceptions in the west part of the country (Pape, Pāvilosta, Rucava) (California Academy of Sciences 2021; Latvijas Dabas Fonds and Latvijas Ornitoloģijas biedrība 2021; Rozenfelde 2018; Spuņģis 2007). The occurrence and distribution of this species in Latvia needs investigation, but it appears, that *T. viridissima* is rather rare.

Additional references:

Albrecht Z (1963) Eesti Sihktiivalised: Orthoptera s. Saltatoria. Eesto NSV Teaduste Akadeemia, Zooloogia ja Botaanika instituut, Tartu, 147 pp. [In Estonian].

Flor G (1860) Die rhynchoten Livlands in systematischer folge beschrieben. Volume 1. Buchdruckerei und Lithographische Anstalt von Carl Schulz, Dorpat, 825 pp. [In German].

Hochkirch A, Willemse LPM, Rutschmann F, Chobanov DP, Kleukers R, Kristin A, Presa JJ, Szovenyi G (2016f) *Stethophyma grossum*. The IUCN Red List of Threatened Species. https://www.iucnredlist.org/species/16084611/74516611 Accessed on: 2021-06-10.

Massa B, Fontana P (2011) Supraspecific taxonomy of Palaearctic Platycleidini with unarmed prosternum: a morphological approach (Orthoptera: Tettigoniidae, Tettigoniinae). Zootaxa 2837 (1): 1-47. https://doi.org/10.11646/zootaxa.2837.1.1

Sardet E, Roesti C, Braud Y (2021) Grasshoppers of Britain and Western Europe. A Photographic Guide. Bloomsbury Publishing, London, United Kingdom, 304 pp.
